# Treatment outcome of tardy ulnar nerve palsy associated with traumatic cubitus valgus by supracondylar shortening wedge rotary osteotomy and ulnar nerve in situ tension release

**DOI:** 10.1186/s12891-022-05324-7

**Published:** 2022-04-20

**Authors:** Chenchen Fan, Maimaiaili Yushan, Yanshi Liu, Yemenlehan Bahesutihan, Kai Liu, Aihemaitijiang Yusufu

**Affiliations:** Department of Trauma and Microreconstructive Surgery, the First Affiliated Hospital of Xinjiang Medical University, Urumqi, Xinjiang China

**Keywords:** Tardy ulnar nerve palsy, Cubitus valgus, Shortening, Rotary, Wedge osteotomy, In situ decompression

## Abstract

**Background:**

Tardy ulnar nerve palsy is a common late complication of traumatic cubitus valgus. At present, the treatment of tardy ulnar nerve palsy associated with traumatic cubitus valgus is still controversial, whether these two problems can be corrected safely and effectively in one operation is still unclear. To investigate the supracondylar shortening wedge rotary osteotomy combined with in situ tension release of the ulnar nerve in the treatment of tardy ulnar nerve palsy associated with traumatic cubitus valgus.

**Methods:**

Between 2012 and 2019, 16 patients who had traumatic cubitus valgus deformities with tardy ulnar nerve palsy were treated with simultaneous supracondylar shortening wedge rotary osteotomy and ulnar nerve in situ tension release. we compared a series of indicators of preoperative and postoperative follow-up for at least 24 months, (1) elbow range of motion; (2) the radiographic correction of the preoperative and postoperative humerus-elbow-wrist angles; (3) the static two-point discrimination and grip strength; and (4) the preoperative and postoperative DASH scores of upper limb function. The minimum follow-up was 24 months postoperative (mean, 33 months; range, 24 ~ 44 months).

**Results:**

The mean ROM was improved from 107 ° preoperatively to 122 ° postoperatively (*P* = 0.001). The mean preoperative elbow wrist angle was 24.6 °, and the mean postoperative humerus-elbow wrist angle was 12.1 ° (*P* < 0.001). The average grip strength and static two-point discrimination improved from 21 kgf and 8 mm to 28 kgf and 4.0 mm (*P* < 0.001 and *P* < 0.001, respectively). The ulnar nerve symptoms were improved in all patients except one. The mean HASH score improved from 29 to 16 (*P* < 0.001).

**Conclusion:**

Supracondylar shortening wedge rotary osteotomy combined with in situ tension release of ulnar nerve is an effective method for the treatment of traumatic cubitus valgus with tardy ulnar nerve palsy, which restored the normal biomechanical characteristics of the affected limb and improved the elbow joint function.

**Supplementary Information:**

The online version contains supplementary material available at 10.1186/s12891-022-05324-7.

## Background

Tardy ulnar nerve palsy is a common late complication of traumatic cubitus valgus deformity caused by nonunion or malunion of distal lateral condyle fracture in children [1–3]. Although various surgical techniques have been proposed for the treatment of posttraumatic cubitus valgus with tardy ulnar nerve palsy, there are few reports on the curative effect of surgical treatment of traumatic cubitus valgus with tardy ulnar nerve palsy. The effect of ulnar nerve postoperative recovery depends not only on the severity of ulnar nerve involvement before the operation but also on whether cubitus valgus deformity is corrected [4–6]. Because serious complications are easy to occur after osteotomy [7], it is generally considered that osteotomy should be considered when the carrying angle is greater than 20° or complicated with serious complications [8]. However, there is little information about supracondylar osteotomy combined with ulnar nerve in situ tension release in patients with traumatic cubitus valgus and tardy ulnar nerve palsy. We believe that while correcting the appearance deformity of cubitus valgus, special attention should be paid to the correction of upper limb biomechanics and abnormal external rotation.

Therefore, we reviewed a group of patients who underwent this combined procedure and compared a series of indicators of preoperative and postoperative follow-up for at least 24 months, (1) elbow range of motion (ROM); (2) the radiographic correction of the preoperative and postoperative humerus-elbow-wrist (HEW) angles; (3) the grip strength and static two-point discrimination; and (4) the preoperative and postoperative DASH scores of upper limb function. To investigate the surgical technique and effect of supracondylar shortening wedge rotary osteotomy combined with in situ tension release of the ulnar nerve in the treatment of tardy ulnar nerve palsy associated with traumatic cubitus valgus.

## Methods

After institutional review board approval, 16 patients with tardy ulnar nerve palsy associated with traumatic cubitus valgus were included in the study between 2013 and 2019. The inclusion criteria included patients with tardy ulnar nerve palsy associated with traumatic cubitus valgus deformity who underwent both supracondylar shortening wedge rotary osteotomy of the distal humerus and ulnar nerve in situ tension release, and postoperative follow-up greater than 24 months. The indications of supracondylar shortening wedge rotary osteotomy for traumatic cubitus valgus deformity are (1) cubitus valgus deformity is more than 20 °; (2) complicated with serious complications such as ulnar nerve palsy.

There were 16 patients with a mean age at the time of surgery of 42 years (range, 17 ~ 55 years) were included in the study (Table 1). There were 14 male patients and 2 female patients. Unfortunately, our department did not accept children’s patients, so there was no study on children’s cases. In the study, traumatic cubitus valgus deformity was secondary to lateral condylar nonunion in 11 cases and lateral condylar malunion in 5 cases. Among the 11 patients with nonunion, 6 cases belonged to Group I nonunion caused by Milch Type I injury, and 5 cases belonged to Group II nonunion caused by Milch Type II injury according to the classification of Toh et al. [2]. All patients had atrophy of internal muscles innervated by ulnar nerve at the initial appearance, which was classified as Dellon grade III [9]. Grade III criteria were defined as patients with persistent paresthesia, abnormal two-point discrimination, measurable weak grip and pinch strength, and intrinsic atrophy. All patients underwent electrical diagnostic tests to confirm the diagnosis of ulnar nerve palsy at the elbow.

**Table 1:** Clinical data of patients

Case	Gender	Age (year)	Followup (months)	HEW angle(°)		ROM(°)		Grip strength (kg)		2-PD (mm)		DASH		Result for deformity correction
				Preoperative	Postoperative	Preoperative	Postoperative	Preoperative	Postoperative	Preoperative	Postoperative	Preoperative	Postoperative	
1	M	28	35	28.8	12.2	90	136	16	20	5	3	23	16	Excellent
2	M	18	28	25.5	11.4	75	110	13	22	14	7	34	18	Excellent
3	M	44	34	28.3	14.6	83	118	20	30	6	4	26	20	Excellent
4	M	17	33	27.3	10.8	112	120	18	23	7	2	25	13	Excellent
5	F	30	42	24.4	13.6	106	112	10	16	10	4	38	21	Excellent
6	M	32	24	23.8	12.4	118	129	13	13	8	4	36	19	Excellent
7	M	46	29	25.2	12.1	113	118	18	25	9	5	25	14	Excellent
8	M	44	38	25.8	16.4	120	126	19	27	8	3	20	15	Excellent
9	M	48	40	20.6	10.7	18	130	30	44	4	4	39	24	Excellent
10	M	56	33	21.8	10.4	120	126	9	16	11	5	40	26	Excellent
11	M	49	30	26.0	13.3	125	129	24	40	9	4	27	20	Excellent
12	M	52	25	21.4	13.1	128	122	33	42	3	5	18	16	Good
13	M	29	44	23.2	11.6	95	116	30	36	7	4	43	20	Excellent
14	M	47	37	24.1	10.4	115	130	27	22	8	5	35	19	Excellent
15	M	50	24	26.2	11.8	96	110	32	39	6	3	24	13	Excellent
16	F	53	36	20.8	9.5	118	124	29	34	11	6	37	22	Excellent

*HEW* humerus-elbow-wrist, *2-PD* two-point discrimination, *M* male, *F* female

**Fig. 1:**
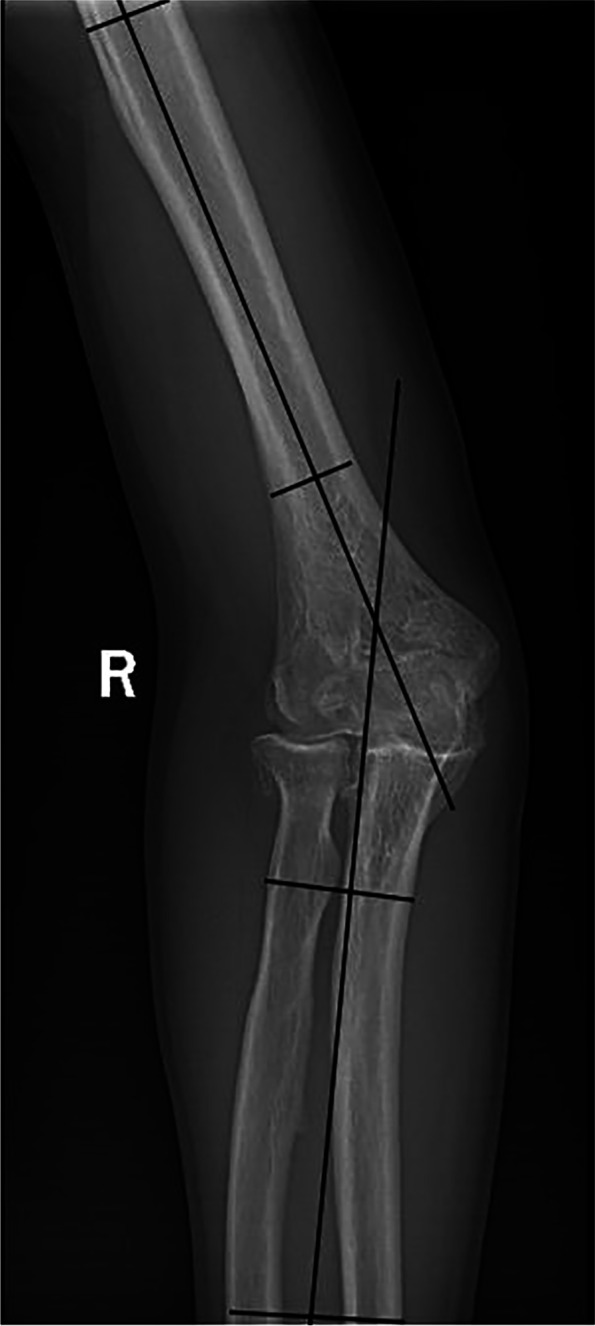
The humerus-elbow-wrist (HEW) angle of the humerus was measured by connecting the midpoint of two lines connecting the medial and lateral cortex of the humerus (proximal and distal) and the midpoint of two lines connecting the medial ulnar cortex and the lateral radial cortex (proximal and distal). The angle between the two lines is the HEW angle

The clinical and radiographic evaluation of the upper extremities was performed before operation. When the elbow joint was extended and the forearm was supinated, the anteroposterior radiographs of both upper extremities were taken, and then the carrying angle is measured on the anteroposterior radiographs [10]. Patients with elbow flexion contracture should keep their medial and lateral condyles at the same level for anteroposterior radiographs to avoid overestimating the HEW angle. To measure the HEW angle, two transverse lines (one proximal and one distal) were drawn on the humerus to connect the medial and lateral cortex, and two transverse lines (one proximal and one distal) were drawn on the forearm to connect the medial ulnar cortex and the lateral radial cortex. Then, draw a line passing through the midpoint of the two lines of the distal and proximal humerus and a line passing through the midpoint of the line of distal and proximal forearm. The intersection of the two lines is measured as the HEW angle (Fig. 1) [7]. To improve the reliability of radiological measurement, we used the average of two values evaluated by different observers. The range of motion of the affected elbow was evaluated and compared with that of the normal elbow. When measuring elbow flexion contracture, the medial and lateral condyles should be kept at the same level, because in patients with cubital valgus, the shoulder joint tends to rotate outward with the extension of the course of the disease, which is easy to cover up the true elbow flexion contracture. The necessary correction angle was estimated between the HEW angle of normal and deformed elbows.

Postoperatively, the patients were followed up at 1 month, 3 months, 6 months, 1 year, and annually thereafter; Standard anteroposterior and lateral radiographs were obtained in each follow-up. Each patient’s HEW angle, elbow range of motion, grip strength, and two-point discrimination were evaluated at 3 months, 6 months, 1 year, and annually thereafter. Each patient also completed the DASH survey at each follow-up in the first year after the operation. The DASH questionnaire included 30 items: 21 questions were used to assess the difficulty of a specific task, 5 questions were used to assess symptoms, and 4 questions were used to assess social function, work function, sleep, and confidence [11]. The DASH score is between 0 and 100, and the higher the score is, the more severe the upper limb disability is. According to Oppenheim’s modified standard, the clinical results of elbow valgus deformity were evaluated by measuring the angle of HEW, ROM of the active elbow, and the presence of complications [12]. The excellent result included that the angle of HEW was 5 ° within the contralateral elbow, the ROM decreased by less than 5 °, and no complication. The good result included that the angle of HEW was 6 ° to 10 ° within the contralateral elbow, the ROM decreased between 6 ° and 10 °, and no complication. The poor result included that the angle of HEW was more than 10 ° of the contralateral elbow, the ROM decreased by more than 10 °, with or without complications.

**Fig. 2:**
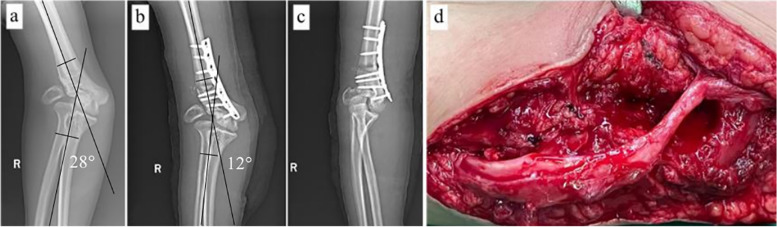
**a** A 28 year old male (patient 1) had cubitus valgus deformity (HEW angle, 28 degrees) and severe tardy ulnar nerve palsy due to traumatic elbow joint injury. **b.c** The supracondylar shortening wedge rotary osteotomy combined with in situ tension release of ulnar nerve was performed. HEW angle was corrected to 12 degrees postoperative, and the effect of deformity correction was excellent. **d** Intraoperatively, the ulnar nerve was variably edematous and pale at the level of the deformity evident

**Fig. 3:**
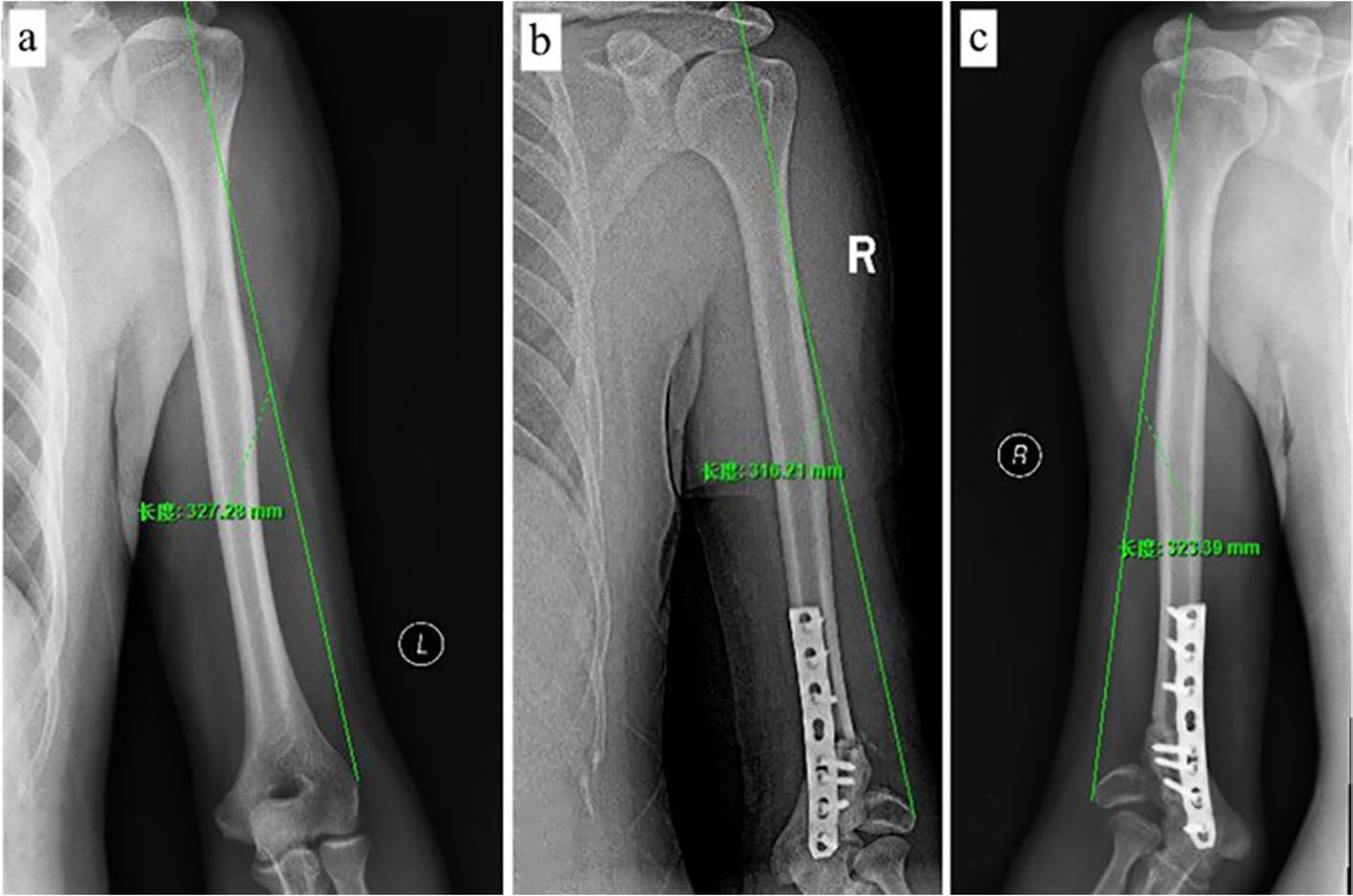
**a** The reference length of the upper arm on the healthy side of patient 1 is about 33 cm. **b.c** Comparing the length of the affected upper arm on the second postoperative day with the one-year postoperative period, it can be seen that the affected arm had increased by another about 2 cm 1 year later, approaching the length of the contralateral limb. The importance of intraoperative shortening of 1-2 cm is seen

Under general anesthesia, the patient was in the supine position. After the application of a sterile tourniquet, a posterior longitudinal incision was made from the distal part of the upper arm to the olecranon. The ulnar nerve was exposed and protected with Penrose drain. Triceps muscle was incised to expose distal humerus and olecranon, and the periosteum was stripped to expose metaphysis and diaphysis of the distal humerus. Osteophytes at the end of the olecranon were removed to promote elbow extension. The wedge osteotomy is simple and easy to determine the osteotomy point. Usually, the distal osteotomy line should be 2 cm above the dorsal attachment of the elbow joint capsule, and the line should be parallel to the horizontal line of the elbow joint. The angle of wedge osteotomy for cubitus valgus was equal to the difference of the HEW angle between the affected side and the normal side (Fig. 2a-c). We shortened the proximal humerus by 1–2 cm to relieve the tension of the forearm extensor muscle and rotated the forearm to the medial side properly to restore the normal structure and upper limb biomechanics (Fig. 3). To avoid obvious bony protrusion on the medial side after osteotomy, the distal bone block can be translated to the outside properly. To make the patients have early rehabilitation training after the operation, the osteotomy should be firmly fixed with a steel plate as far as possible after satisfactory reduction.

The ulnar nerve was decompressed in situ after the correction of elbow angle deformity. Abduction and external rotation of the affected limb. A medial skin flap was retracted to expose the ulnar nerve completely, which is macroscopically seen to be variably edematous and pale in the valgus deformity (Fig. 2d). The arcuate ligament was carefully dissected from the olecranon attachment and turned to the medial epicondyle of the humerus. The ulnar nerve was separated and pulled forward for protection to expose the ulnar nerve sulcus. The periosteum was carefully dissected from the attachment on the humeral condyle and turned to the posterolateral side of the humerus for protection, to be used as a reserve for the grooved bed of the ulnar nerve. The osteophyte in the ulnar nerve sulcus was removed and the ulnar nerve sulcus was appropriately expanded and deepened with a grinding drill. After cleaning the bone fragments to prevent heterotopic ossification and fixing the stripped periosteum back to its original position, the ulnar nerve was placed back into the ulnar nerve groove. Whether the release of the epineurium of the ulnar nerve in such patients should depend on the intraoperative situation. Clinically, it is found that most of the ulnar nerve in patients with Dellon grade III of cubital tunnel syndrome have obvious pathological changes, such as bulging and hyperplasia of the epineurium (Fig. 2d). Therefore, we believe that after relieving abnormal tension and compression, it is necessary to perform the release of the epineurium of the ulnar nerve under the microscope in most cases. In general, ischemic traction injury of nerve does not need to release the endoneurium unless external neurolysis and wedge osteotomy fail to solve the abnormal tension. Passively move the elbow joint to ensure that there is no abnormal tension, slippage, or compression of the ulnar nerve, and then suture the arcuate ligament back to its original position. Close the incision layer by layer, bend the elbow joint 90 degrees with a long arm splint for 2 weeks, and then perform ROM training passively and actively.

SPSS Version 21.0 (SPSS, Inc., Chicago, IL, USA) was used for statistical analyses. HEW angles, elbow ROM, grip strength, 2-point discrimination, and DASH score before surgery were compared with the corresponding values at the last follow-up using the Wilcoxon signed-rank test. A *p*-value less than 0.05 was considered significant.

## Results

Before surgery, the average active flexion of these patients was 107 °(95% CI, 99 ° – 116 °); after surgery, the average active flexion was 122 ° (95% CI, 118 ° – 127 °) (*p* = 0.001). However, one patient lost 6 ° ROM. According to Oppenheim’s improved standard, 15 patients had excellent correction effect, and one patient had a good correction effect (Table 1). In addition, elbow extension function did not show significant statistical difference before and after surgery. At 3 months after the operation, all distal humeral osteotomy sites showed healing. The average HEW angle was corrected from 24.6 ° (95% CI, 23.2 ° – 25.9 °) to 12.1 °(95% CI, 11.2 ° – 13.1 °) (*p* < 0.001), and the correction effect of cubitus valgus before and after operation is shown in Fig. 2a-b.

At the last follow-up, the grip strength increased from a mean of 21 kg of force (95% CI, 17–26 kg of force) to 28 kg of force (95% CI, 23–33 kg of force) (*p* < 0.001). The mean 2-point discrimination improved from 8 mm (95% CI, 6–9 mm) to 4 mm (95% CI, 3–5 mm) (*p* < 0.001). The mean DASH score improved from 31 points (95% CI, 26–35 points) to 19 points (95% CI, 16–20 points) (*p* < 0.001). All but one patient reported subjective improvement in their symptoms.

## Discussion

Whether supracondylar osteotomy of the distal humerus should be simultaneously combined with ulnar nerve in situ tension release for the treatment of tardy ulnar nerve palsy associated with cubitus valgus remains controversial. We evaluated a group of patients who underwent this combined operation at the same time, rather than in stages, and evaluated imaging and clinical outcomes for at least 2 years. The results showed that the symptoms and signs of ulnar nerve were improved, the deformity was well corrected, and the function of the elbow joint was not decreased.

In our study, we used supracondylar shortening wedge rotary osteotomy to correct traumatic cubitus valgus. The patients were followed up for at least 2 years. Although the elbow range of motion on the affected side could not be recovered completely, its function was improved compared with that before operation. We used two conventional reconstruction plates or LCP reconstruction plate fixation, which can stabilize the osteotomy so that the elbow joint can get early exercise. We believe that removal of the osteophyte at the tip of the olecranon through posterior capsulotomy is one of the reasons for the increased active elbow extension in our patients. Moreover, the treatment of old nonunion of lateral condyle of the humerus is still controversial [3, 13]. In the study, none of the 11 patients with nonunion of the lateral condyle of the humerus underwent osteosynthesis, this seems to be another reason why our patients with lateral condylar nonunion maintained their elbow ROM postoperatively. Supracondylar osteotomy is usually accompanied by severe complications, including loss of elbow range of motion, lateral condylar fracture necrosis, and persistent nonunion [7, 14]. Therefore, the indication of osteotomy should be considered in patients with elbow pain, limited movement, and tardy ulnar nerve palsy, and other serious complications [12].

All normal HEW angle was obtained in all patients without recurrence of deformity or residual eminence of the medial condyle. Although there is no consensus, for patients with an angle below 20°, we do not recommend osteotomy as a priority because it is prone to serious complications after osteotomy. If the angle exceeds 20°, supracondylar osteotomy of the distal humerus is usually indicated [8]. Because of the advantages of simplicity and easy implementation, the medial closing-wedge osteotomy is traditionally used to correct cubitus valgus, however, it has been reported that some patients failed to correct deformity, reduced range of motion of the elbow, and other complications postoperative. Clinically, We found that in patients with cubitus valgus, the length of the humerus on the affected side is longer than that on the healthy side. Studies have shown that the length of the humerus after supracondylar fracture of the distal humerus increases by 1% of the original length on average even with effective internal fixation [15]. However, the mechanism of the increase of the length of the affected limb after the fracture of the lateral condyle of the humerus still needs further research, and there is only a few literature support at present. Therefore, we believe that the abnormal increase of humeral length will be more in patients with traumatic cubitus valgus, and long-term cubitus valgus deformity leads to contracture of the extensors of the forearm. The abnormal traction of contracture forearm extensor muscle leads to an abnormal increase of valgus force after the traditional medial closing-wedge osteotomy, which hinders the fixation needed to maintain the correction and easily leads to the failure of fixation and the joint force imbalance. According to this, we routinely shortened the osteotomy surface of proximal humerus by 1 to 2 cm after the osteotomy. In addition, we should pay attention to the changes in normal structure and biomechanics during the development of traumatic cubitus valgus. Due to the traction of forearm extensor muscles, the distal humerus rotated laterally during healing. Therefore, in the process of osteotomy to correct the deformity, the distal humerus should be rotated medially according to the actual situation to restore the normal structure and biomechanics as much as possible [16], to increase the range of motion of the elbow joint and reduce the postoperative complications.

Traumatic cubitus valgus deformity can lead to tardy ulnar nerve palsy [2, 17]. Nerve sonography showed that the cubitus volume of cubitus valgus deformity decreased, echo of ulnar nerve decreased, nerve swelling was obvious, and local blood flow sometimes increased. Anterior transposition of the ulnar nerve has become the mainstream surgical treatment for tardy ulnar nerve palsy caused by traumatic cubitus valgus deformity [8]. Although this surgical scheme completely decompresses the ulnar nerve and solves the traction and friction of the ulnar nerve during elbow flexion, there are many disadvantages in this surgical method, such as dissociating at least 10 cm ulnar nerve to meet the requirements of anterior transposition, Extensive dissociation increases the injury and does not rule out the possibility of injuring the flexor carpal ulnar muscle and affecting the blood supply of ulnar nerve [18]. Moreover, the fixation time of the elbow joint is long after the operation, the ulnar nerve is mostly located under the soft tissue, the nerve without cubital tunnel protection is easily damaged by an external force, and the incidence of complications such as long-term scar compression is high. Given the prerequisite of traumatic cubitus valgus and the anatomical characteristics of the abnormal prominent medial condyle, the ulnar nerve groove can be expanded appropriately after releasing the ulnar nerve. During the operation, we first perform osteotomy to correct cubitus valgus deformity, and then release the ulnar nerve. Its advantage is that the abnormal tension of the nerve is solved after osteotomy, which can determine the specific position and length of the ulnar nerve to be released, so as to avoid excessive release and secondary injury. Moreover, it is more convenient to operate the ulnar nerve after the abnormal tension is solved. Of course, it is necessary to dissect and protect the ulnar nerve before osteotomy to avoid unnecessary injury. By expanding the four walls of the cubital canal, the depth and width of the ulnar nerve groove can be expanded, and the instability and dislocation of the ulnar nerve can be solved without changing the normal anatomical structure and course of the ulnar nerve, It avoids the nerve pain and injury caused by an external force. At the same time, it also solves the problem of abnormal reduction of cubital tunnel volume during elbow flexion, which effectively avoids the extreme increase of ulnar nerve internal pressure when elbow flexion, greatly improving ulnar nerve microcirculation and avoiding ulnar nerve injury or aggravation due to ischemia and hypoxia. The combined operation is simple, the trauma is small, the blood supply of the ulnar nerve is not damaged, the release is complete, the curative effect is remarkable, and the recurrence rate of ulnar neuritis is reduced [19]. In the current study, the mean DASH score increased significantly from 29 to 16 at 2 years after surgery (*P* = 0.001). In addition, we believe that both biomechanical recovery and ulnar nerve symptoms will improve the strength of hand muscles, but which factor is dominant still needs further research. It seems to be a promising clinical outcome that supracondylar shortening wedge rotary osteotomy combined with in situ tension release of the ulnar nerve can reduce pathological traction of ulnar nerve at the elbow.

The study also has several limitations. The data were collected retrospectively, including only a few patients. In addition, no control group was treated with in situ tension release of the ulnar nerve. All of our patients had a cubitus valgus deformity greater than 20° and underwent correction of the deformity concurrent with ulnar nerve in situ tension release if they wanted. However, few cases require osteotomy and ulnar nerve release in situ at the same time in clinical practice. Patients with elbow flexion contracture tend to rotate the shoulder externally to cover up the flexion contracture. This compensatory movement of the shoulder may overestimate the angle of the HEW. So we need to instruct our patients how to keep their medial and lateral condyles at the same horizontal plane to obtain the correct elbow projection during radiography.

In addition, there is no reliable, reproducible, and effective prognostic indicator for ulnar nerve palsy caused by traumatic cubitus valgus deformity treated by both supracondylar correction osteotomy and ulnar nerve in situ release. The DASH score is a general upper limb function scoring tool. We used it to evaluate the disability of traumatic cubitus valgus deformity. Although retrospective studies usually have selection bias, our study does not have this problem because we performed deformity osteotomy and ulnar nerve in situ tension release simultaneously in all patients. One of our patients was excluded from this study due to a follow-up of fewer than 2 years. The patient’s last visit was 6 months after surgery and the osteotomy site was completely healed at that time. However, the assessment at that time did not seem to be sufficient to judge the outcome of the operation. Therefore, we analyzed the data of 16 patients who were followed up for more than 2 years.

Based on the results of this small retrospective study, we are cautiously optimistic about supracondylar shortening wedge rotary osteotomy combined with in situ tension release of the ulnar nerve for traumatic cubitus valgus with tardy ulnar nerve palsy. This combined operation can significantly improve the function and appearance of the elbow joint and the symptoms of ulnar nerve palsy caused by long-term traction and friction, and effectively prevent ulnar nerve slippage caused by instability of the elbow joint, It is a surgical scheme with anatomical characteristics, simple operation, less trauma, and satisfactory curative effect.

## Conclusion

Supracondylar shortening wedge rotary osteotomy combined with in situ tension release of ulnar nerve is an effective method for the treatment of traumatic cubitus valgus with tardy ulnar nerve palsy, which restored the normal biomechanical characteristics of the affected limb and improved the elbow joint function.

## Supplementary Information

**Additional file 1.**

## Data Availability

The datasets analyzed during the current study are available from the corresponding author on reasonable request.

## Abbreviations

HEW: Humerus-elbow-wrist
2-PD: Two-point discrimination
M: Male
F: Female
